# Systematic Review and Meta-Analysis of Randomized Controlled Trials of Liangxue Tongyu Formula on Patients With Acute Intracerebral Hemorrhage

**DOI:** 10.3389/fphar.2020.00437

**Published:** 2020-04-15

**Authors:** Chao Jiang, Xiaojuan Yang, Ju Dong, Guochun Li

**Affiliations:** Department of Public Health, Nanjing University of Chinese Medicine, Nanjing, China

**Keywords:** acute intracerebral haemorrhage, Liangxue Tongyu formula, systematic review, meta-analysis, randomized controlled trials (RCT), traditional Chinese medicine

## Abstract

**Background:**

As a traditional Chinese medicine (TCM) prescription for acute stroke, Liangxue Tongyu formula (LXTYF) was widely used as auxiliary treatment measure in some clinical practice. This study aimed to evaluate the clinical efficacy and safety of LXTYF combined western conventional medicine (WCM) with WCM only for acute intracerebral hemorrhage (ICH).

**Methods:**

We systematically searched PubMed, Embase, Cochrane Library, CMB (Chinese biomedicine database), CNKI (China National Knowledge Infrastructure), WanFang, and VIP until August 2019 to confirm relevant randomized controlled trials (RCTs) compared the combination of LXTYF and WCM with WCM alone for the treatment of acute ICH. Two investigators independently assessed the risk of bias, and extracted and analyzed the data from the identified studies using RevMan 5.3.0 software following Cochrane’s standard and PRISMA guidelines. The herbal compositions of LXTYF were also assessed.

**Results:**

15 RCTs were identified, totally recruiting 1648 patients with acute intracerebral hemorrhage. Compared with the WCM alone, the combination therapy of LXTYF with WCM could improve the clinical effective rate (*RR*, 1.21; 95% CI, 1.15–1.25, *P* < 0.05) and ADL score (MD, 18.09; 95% CI, 12.11–24.07; *P* < 0.05), and reduce syndrome scores of the TCM (MD, −4.11; 95% *CI*, −4.69 to −3.53; *P* < 0.05) and the Glasgow outcome score(GOS) (*MD*=0.43, 95%*CI*: 0.06 to 0.79, *P*=0.02) Moreover, there was no sufficient evidence to indicate the adverse effects would increase compared with WCM alone.

**Conclusion:**

Based on current evidence, we concluded that the combined therapy had some benefits in treating acute intracerebral hemorrhage. However, considering the potential biases and limitations of our study, additional large, high-quality RCTs are required in the future to confirm or refute the effects of LFTYF combined with WCM in acute stroke.

## Introduction

Intracerebral hemorrhage (ICH) is the non-traumatic hemorrhage caused by rupture of blood vessels in brain parenchyma, which is one of most fatal subtypes of stroke (Wang and Talked, 2009). It has attracted board attention because of its high morbidity and mortality worldwide (Adeoye and Broderick, 2010; Luo, 2010). Especially in Chinese population, the overall incidence of stroke and the proportion of ICH is higher than white population (Tsai et al., 2013). Acute phase refers to the first 3 weeks of illness. In this period, the brain tissue is damaged and has severe cerebral edema, brain dysfunction and the body is in stress state. The death rate of the acute stage is 30% to 40% (Qureshi et al., 2009). In routine clinic, the treatment of acute ICH mainly adopts the western conventional medicine (WCM), such as preventing further bleeding, controlling brain edema, decreasing intracranial pressure, maintaining life function, and preventing complications (Dimitre et al., 2010). Obviously, the actual efficacy of the WCM alone does not meet expectations (Andaluz and Zuccarello, 2009; Zhou et al., 2014). Therefore, new treatment drugs need to be developed under the guidance of evidence-based medicine. Several studies reported that traditional Chinese medicine (TCM) was benefit for stroke treatment (Li et al., 2015). As a classical TCM prescription for acute ICH, Liangxue Tongyu formula (LXTYF), originating from ‘Xi Jiao Di Huang Tang’ recorded in the ancient medical book ‘Bei Ji Qian Jing Yao Fang’ of AD 652, was used as auxiliary treatment measure in some clinical practice in China. But there is no systematic review to evaluate the quality of these studies and synthesize evidence on the effects of this formula. Therefore, the objective of this article is to systematically collect randomized controlled trials (RCTs) to evaluate the efficacy and safety of LXTYF in the treatment of acute ICH.

## Materials and Methods

This systematic review and meta-analysis was performed following the guidelines of PRISMA (the Preferred Reporting Items for System Reviews and Meta-Analysis) (Moher et al., 2015) and was registered in PROSPERO. The registration identifier of the protocol was CRD42018093324 (Jiang et al., 2018).

### Types of Studies

All clinical RCTs adopting WCM combined with LXTYF to treat acute ICH were included, which was not limited to publish language, publish form, and whether to adopt blind method.

### Types of Participants

The patients with ICH were included according to the diagnosis criteria of western medicine: Diagnostic points of various cerebrovascular diseases revised at the Fourth National Conference of the China Society of Medicine on Cerebrovascular Diseases in 1995 (Chen, 1996). The diagnosis criteria of stroke in Chinese medicine are as follows: Evaluation criteria for diagnosis and efficacy of apoplexy established by State Administration of Traditional acute encephalopathy research collaborative groups in 1996 (The State Administration of Traditional acute encephalopathy research collaborative groups, 1996). Patients who had the syndromes of TCM in fengyang, fire-heat, blood stasis, stagnant heat, phlegm-heat, yin-deficiency, and yang-predominance were also included.

### Types of Interventions

Eligible comparisons were LXTYF+WCM versus the WCM alone. There was no limitation on the dosages or dosage form (oral medicine or injection) or treatment courses. The control groups were adopted WCM, such as decreasing intracranial pressure, regulating blood pressure, keeping water electrolyte balance, preventing the occurrence of stress ulcers, anti-infection, and other symptomatic treatment. The treatment groups were adopted LXTYF combined with WCM, whether it was an oral agent or an injections. The formula of prescription mainly contains six ingredients: Rheum officinale Baill 10 g, Bubali cornu 30 g, Rehmannia glutinosa Libosch 20 g, Paeonia lactiflora Pall 15 g, Paeonia suffruticosa Andrews 10 g, Acoru tatarinowii Schott 10 g.

### Outcome Indicator

With reference to the diagnostic scores of stroke in evaluation criteria for diagnosis and efficacy of apoplexy (The State Administration of Traditional acute encephalopathy research collaborative groups, 1996). To evaluate the therapeutic effect by the percentage of TCM syndrome scoring improving(S) and the TCM syndrome scoring after treatment (F) of the patients. S≥81%, F≤6 Basic recovery; 81% > S≥56% Significant progress; 56% > S≥36% Progress; 36% > S≥11% Slight progress; 11% > S≥0% No change; S < 0% deterioration. Total efficiency = (basic recovery + significant progress + progress + slight progress)/*n*×100%. The primary outcome was clinical total effective rate after the treatment. Other assessment outcomes also included volume of cerebral hematoma and brain edema, Glasgow outcome scale (GOS), Activities of daily living(ADL) score, TCM syndrome score, and number of adverse events.

### Exclusion Criteria

The following studies were excluded: (1) the full text was not available through electronic retrieval, manual retrieval, and the authors’ email; (2) repetitive publication; (3) Targeted interventions were not LXTYF combined with WCM. (4) Although it reported to use LXTYF, the composition has significant difference. (5) Data was incorrect, incomplete or unavailable. (6) The patient with intracranial hemorrhage caused by transient ischemic attack, cerebral infarction, subarachnoid hemorrhage, blood disease, tumorous trauma, with cerebral hernia or deep coma, had serious complication in heart, lung, liver, kidney; had the syndrome of Chinese medicine in qi-deficiency, phlegm-dampness, prostration syndrome. (7) Reviews or meta-analysis, retrospective studies, case reports, experimental research, and conference abstracts. (8) RCTs with wrong allocation sequence generation method. For example, the patients enrolled into the different group according to their will.

### Search Strategy and Methods for Identification of Studies

Two independent researchers (CJ and XY) searched the electronic databases thoroughly including PubMed, EMBASE, the Cochrane Library, CNKI, the Wanfang database, and VIP Journals Database from inception to March 2018. The following multiple combinations of search keywords were used: (Liangxue Tongyu formula) AND (cerebral hemorrhage OR stroke OR cerebral apoplexy OR hemorrhagic stroke) for English databases and (Liang xue tong yu (the Yu have two different Chinese characters)) AND (Chu xue zhong feng OR Nao chu xue OR Nao zu zhong) in Chinese phonetic alphabets for Chinese database. In order to avoid omission, the references to the articles and reviews we retrieved were also examined. Publication date or country were not limited.

### Literature Selection and Data Extraction

After literatures duplicate checking, two reviewers (CJ and XY) screened all articles according to the inclusion/exclusion criteria, extracted the data and evaluated the risk of bias, independently. Then checked each other. Disagreement were resolved through discussion with a third party (GL). The extracted data mainly included: author names, publication year, study design, sample size, detail of intervention: names, dosage form, dosage, ingredient, treatment courses, outcomes, and quality assessment.

### Risk of Bias Assessment

Two researchers (CJ and XY) independently assessed the risk of bias in identified RCTs with the reference to risk of bias tool of Cochrane Collaboration (Higgins and Green, 2011). The assessment tool of risk of bias contains the following evaluation contents: random sequence generation; allocation concealment; blinding; incomplete data; selective reporting; and other bias. According to the detailed rules, each item could be divided into high risk, low risk, unclear risk. When there was no enough information to determine whether a study satisfies the scoring criteria, the study was considered as an unclear risk. If there was a difference in the judgement between two researchers, it would be settled by discussion with a third party.

### LXTYF Composition

The frequency of most commonly used ingredients of LXTYF was calculated, and those herbs or ingredients were described in detail.

### The Quality of the Included RCT

In order to assess the quality of the included RCTs, we used a rating system (Wang et al., 2019) as follows: (1) high quality, full information about the botanical material is provided, including a voucher specimen; (2) moderate quality, only partial information about the botanical material is provided, and a voucher specimen is missing; there are taxonomic inaccuracies; (3) low quality, inadequate information and overall taxonomically is inadequate.

### Statistics Analysis

Review Manager 5.3 (Cochrane, 2012) was used to merge the outcomes of clinical trials and analysis data. Quantitative outcomes were expressed as weighted mean difference (WMD), while qualitative outcomes were expressed as risk ratio (*RR*). Both 95% confidence interval was also calculated. Chi-square test and I-square (*I*^2^) index were used to test heterogeneity. The result were considered as having high heterogeneity if *P* value of Chi-square test less than 0.05 and *I*^2^ values greater than 50%. At this time, the random effects model was used for analysis, otherwise the fixed effects model was adopted. Publication bias was inspected by using funnel plots.

## Results

### Procedure for Study Selection

**Figure 1:**
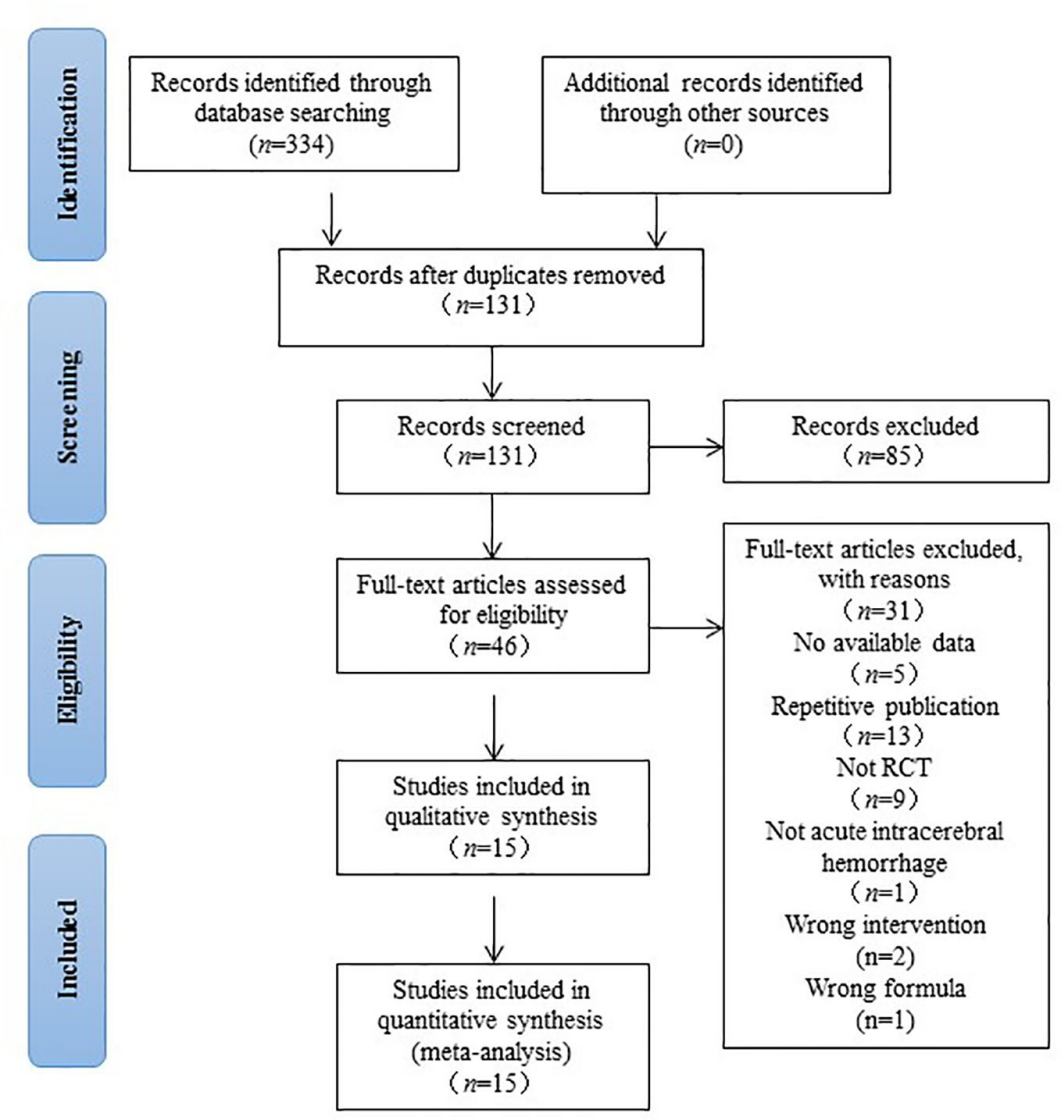
Preferred reporting items for systematic reviews and meta-analysis diagram of searching.

After a comprehensive searched in multiple databases we initially identified 334 studies. After deleting the duplicate articles, 131 studies were retained for further confirmation. By reading the titles and abstracts, 85 studies that did not conform to inclusion criteria were eliminated. The remaining 46 studies were read in full texts, and 31 studies were ultimately excluded by the following reasons: (1) the article was a review or theoretical summary; (2) not a clinical trial; (3) no available data; (4) repetitive publication; (5) not acute ICH; (6) wrong comparison; (7) wrong formula. The screening procedure was illustrated in **Figure 1**.

### Characteristics of Included Studies

This systematic review eventually included 15 studies published in Chinese from 2000 to 2019. The sample size varied from 58 to 337, with a total of 1648 patients with acute ICH (823 patients were in experimental group and 825 patients were in control group). The course of treatment lasted from 14 d to 28d in 15 studies. The characteristics of the 15 RCTs were summarized in **Table 1**. Of the 15 studies, 1 study compared Liangxue Tongyu injection plus WCM versus WCM alone (Gu et al., 2007). The remaining studies were all compared decoction of LXTYF plus WCM with WCM alone. One RCT (Gu et al., 2007) used a commercial preparation. In the other 14 RCTs (Fan et al., 2000; He, 2018), the preparations were made in hospital including the associated pharmaceutical quality control. The ingredients of the prescription and pharmaceutical quality control in each included RCT was illustrated in detail in **Table 2**.

**Table 1:** The characteristics of included studies.

Included trials	Publication language	Study design	Eligibility criteria	No. of participants (male/female); mean age (years)		Interventions		Dosing regimen	Course of treatment	Outcome index	Intergroup differences
				Trial	Control	Trial	Control				
Fan et al., 2000	Chinese	RCT, single center	CMAN standard	32(19/13) 64.44 ± 12.12	32(17/15) 61.31 ± 11.91	LXTYF+WCM	WCM	30ml/t bid	4w	mortality clinical total effective rate the activity of daily living scale(ADL) volume of hematoma volume of encephaledema side effects and complication	*P* > 0.05 *P* < 0.05 *P* < 0.01 *P* > 0.05 *P* < 0.01
Gu et al., 2007	Chinese	RCT, single center	ECCSTCM standard	32(19/13) 56.2	32(20/12) 59.4	LXTYF+WCM	WCM	20ml/t bid	2w	clinical total effective rate mortality signs of the nervous system	*P* < 0.01 *P* > 0.05 *P* < 0.01
Sun, 2011	Chinese	RCT, single center	ECCSTCM standard	31	31	LXTYF+WCM	WCM	One dose/d bid	3w	clinical total effective rate	*P* < 0.05
				(41/21)							
				54.2 ± 2.1							
Guo et al., 2012	Chinese	RCT, single center	ECCSTCM standard	168(120/4) 63.4 ± 11.82	169(98/71) 63.95 ± 11.63	LXTYF+WCM	WCM	One dose/d bid	3w	clinical total effective rate TCM syndrome scoring volume of hematoma grading of cerebral edema GOS	*P* < 0.01 *P* < 0.05 *P* > 0.05 *P* > 0.05 *P* < 0.05
Zhao, 2012	Chinese	RCT, single center	ECCSTCM standard	36	36	LXTYF+WCM	WCM	One dose/d bid	3w	clinical total effective rate	*P* < 0.05
				(48/24)							
				61.3 ± 10.8							
Li L, 2013	Chinese	RCT, single center	ECCSTCM standard	56(39/17) 51.96 ± 10.95	57(38/19) 52.19 ± 11.26	LXTYF+WCM	WCM	One dose/d NR	4w	clinical total effective rate TCM syndrome scoring	*P* < 0.05 *P* < 0.05
Li X, 2013	Chinese	RCT, single center	ECCSTCM standard	112 NR	112 NR	LXTYF+WCM	WCM	One dose/d qd	3w	clinical total effective rate TCM syndrome scoring GOS	*P* < 0.01 *P* < 0.01 *P* < 0.01
Gai, 2014	Chinese	RCT, single center	ECCSTCM standard	40(23/17) 64 ± 7	40(22/18) 64 ± 7	LXTYF+WCM	WCM	One dose/d bid	3w	clinical total effective rate TCM syndrome scoring score of stagnant heat blocking aperture 4. side effects and complication	*P* < 0.05 *P* < 0.05 *P* < 0.05
Suo et al., 2014	Chinese	RCT, single center	ECCSTCM standard	80(45/35) 65.1 ± 4.2	80(44/36) 64.9 ± 4.5	LXTYF+WCM	WCM	One dose/d NR	4w	clinical total effective rate	*P* < 0.05
Fan et al., 2016	Chinese	RCT, single center	ECCSTCM standard	67(48/19) 64.58 ± 12.19	67(44/23) 62.75 ± 11.81	LXTYF+WCM	WCM	One dose/d bid	3w	clinical total effective rate TCM syndrome scoring volume of hematoma	*P* < 0.05 *P* < 0.05 *P* > 0.05
Li and Luo, 2016	Chinese	RCT, single center	ECCSTCM standard	33(18/15) 55.9 ± 4.7	32(17/15) 56.2 ± 4.6	LXTYF+WCM	WCM	One dose/d NR	3w	clinical total effective rate TCM syndrome scoring	*P* < 0.05 *P* < 0.05
Wang, 2016	Chinese	RCT, single center	ECCSTCM standard	33(20/13) 54.2 ± 2.7	33(18/15) 55.3 ± 2.5	LXTYF+WCM	WCM	One dose/d bid	4w	clinical total effective rate	*P* < 0.05
Han, 2017	Chinese	RCT, single center	ECCSTCM standard	33	33	LXTYF+WCM	WCM	One dose/d bid	3w	clinical total effective rate	*P* < 0.05
				(38/28)							
				61.5 ± 10.6							
He, 2018	Chinese	RCT, single center	ECCSTCM standard	42(23/19) 70 ± 10.84	42(20/22) 70.1 ± 11.01	LXTYF+WCM	WCM	One dose/d bid	2w	clinical total effective rate TCM syndrome the activity of daily living scale(ADL) NIHSS side effects and complication	*P* < 0.05 *P* < 0.05 *P* < 0.05 *P* < 0.05
Kong, 2018	Chinese	RCT, single center	ECCSTCM standard	29(20/9) 56.34 ± 6.13	29(19/10) 56.17 ± 6.27	LXTYF+WCM	WCM	One dose/d bid	3w	clinical total effective rate TCM syndrome volume of hematoma recurrence rate	*P* < 0.05 *P* < 0.05 *P* < 0.05 *P* < 0.05

RCT, randomized controlled trial; CMAN, Chinese medical association of neurosurgery; ECCSTCM, encephalopathy committee of the Chinese society of traditional Chinese medicine; LXTFY, Liangxue Tongyu formula; WCM, Western conventional medicine; d, day; bid, twice per day; qd, once per day; NR, not reported; w, week.

**Table 2:** Ingredient, preparation quality control of the LXTYF in included studies.

Included trials	Prescription name	Ingredients of herb prescription	Preparations	Quality control	Chemical analysis reported? (Y/N)	Botanical material information	Voucher specimen	Quality
Gu et al. (2007)	LXTYF	Rheum officinale Bail, Rehmannia glutinosa Libosch, Bubalus bubalis Linnaeu, Paeonia suffruticosa Andr, Acortw tatarinowii Schott, Parmx notoginseng, Gardenia jasminoides Ellis	Injection	Traditional Chinese patented medicine WY: Z20050071	Y-HPLC	P	+	High
Fan et al. (2016)	LXTYF	Rheum officinale Bail, 10 g; Rehmannia glutinosa Libosch, 20 g; Bubali cornu, 30 g; Paeonia suffruticosa Andr, 10 g; Paeonia lactiflora Pall, 15 g; Acortw tatarinowii Schott, 10 g;	Decoction	Hospital preparation	N	P	−	Moderate
Guo et al. (2012)	LXTYF		Decoction	Hospital preparation	N	P	−	Moderate
Li X. (2013)	LXTYF		Decoction	Hospital preparation	N	P	−	Moderate
Wang (2016)	LXTYF		Decoction	Hospital preparation	N	P	−	Moderate
Zhao (2012)	LXTYF		Decoction	Hospital preparation	N	P	−	Moderate
Suo et al. (2014)	LXTYF		Decoction	Hospital preparation	N	P	−	Moderate
Sun (2011)	LXTYF		Decoction	Hospital preparation	N	P	−	Moderate
Han (2017)	LXTYF		Decoction	Hospital preparation	N	P	−	Moderate
Kong (2018)	LXTYF		Decoction	Hospital preparation	N	P	−	Moderate
Gai (2014)	LXTYF	Rheum officinale Bail, 10 g; Rehmannia glutinosa Libosch, 30 g; Bubalus bubalis Linnaeu, 30 g; Paeonia suffruticosa Andr, 15 g; Paeonia lactiflora Pall, 15 g; Acortw tatarinowii Schott, 12 g	Decoction	Hospital preparation	N	P	−	Moderate
He (2018)	LXTYF	Rheum officinale Bail, 10 g; Rehmannia glutinosa Libosch, 10 g; Bubalus bubalis Linnaeu, 30 g; Paeonia suffruticosa Andr, 10 g; Paeonia lactiflora Pall, 10 g; Acortw tatarinowii Schott, 10 g	Decoction	Hospital preparation	N	P	−	Moderate
Li L. X. (2013)	LXTYF	Rheum officinale Bail, 12 g; Rehmannia glutinosa Libosch, 21 g; Bubalus bubalis Linnaeu, 24 g; Paeonia lactiflora Pall, 24 g; Acortw tatarinowii Schott, 12 g	Decoction	Hospital preparation	N	P	−	Moderate
Li and Luo (2016)	LXTYF		Decoction	Hospital preparation	N	P	−	Moderate
Fan et al. (2000)	LXTYF	Rheum officinale Bail, 10 g; Rehmannia glutinosa Libosch, 15 g; Prunus persica Batsch, 10 g;	Decoction	Hospital preparation	N	P	−	Moderate

LXTFY, Liangxue Tongyu formula; UR, unreported; Y, yes; N, no; HPLC, high-performance liquid chromatography; F, full information about the botanical material is provided; P, partial information about the botanical material is provided; I, Inadequate information about the botanical material is provided; +, included a voucher specimen; －, a voucher specimen is missing.

### The Reporting Completeness of the Material in Included Studies

We accessed the reporting completeness of the material in each study with a rating system, which is related to the information about the botanical material and voucher specimens. Only one RCT (Guo et al., 2012) are of high quality, which provided the full information about the botanical material and included voucher specimens. The remaining 14 RCTs are of moderate quality, which provided partial information about botanical material and did not provided voucher specimens. We confirmed that only one RCT (Guo et al., 2012) did chemical analysis though high performance liquid chromatography (HPLC). The remaining RCT did not report the relevant information. However, our team did the same analysis in LXTYF and reported in previous articles (Li et al., 2018). Meanwhile, we also report the results in the **Supplementary Material**. The detail was summarized in **Table 2**.

### Risk of Bias and Quality of Included Studies

**Figure 2:**
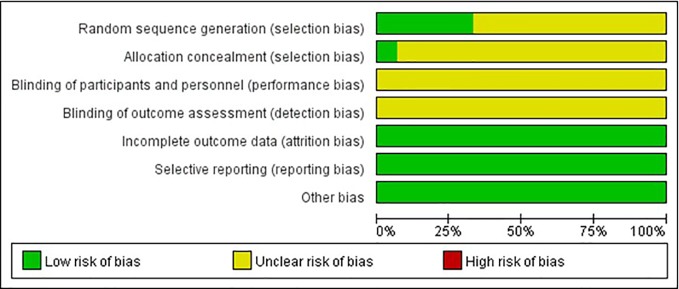
Risk of bias graph.

**Figure 3:**
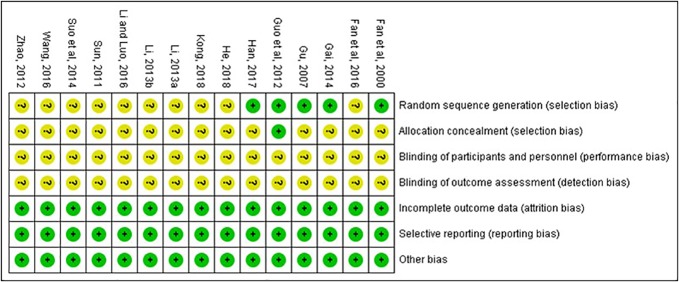
Risk of bias summary.

**Figure 4:**
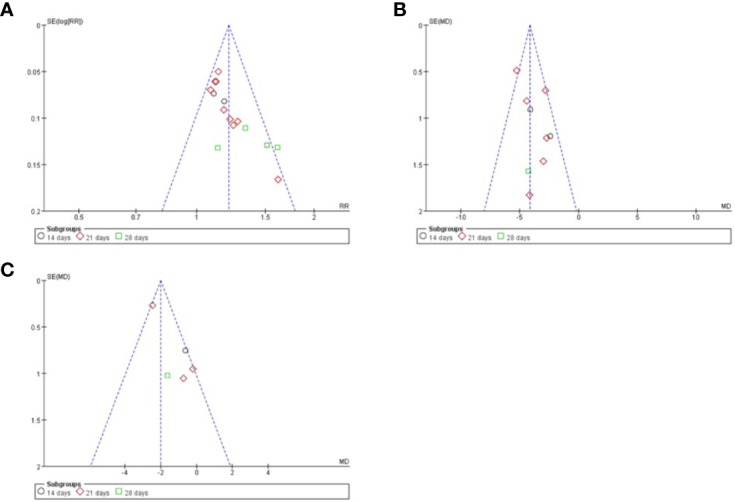
Funnel plots of different index. **(A)** clinical total effective rate; **(B)** TCM syndrome score; **(C)** volume of cerebral hematoma.

The quality of the included studies was also evaluated according to the risk of bias assessment tool of standard RCTs evidence recommended by Cochrane Collaboration. One study used computer to generate random number for random allocation (Guo et al., 2012) and two studies used paired randomization (Fan et al., 2000; Gu et al., 2007). Two studies used random number table (Gai, 2014; Han, 2017). All these five studies were regarded as low risk of bias, and the others claimed that they used randomization but did not report the details of how to randomize, which we considered to have unclear risk of bias. Except Guo’s research, none of the other studies mentioned method of allocation concealment (Guo et al., 2012). Besides, none of the included RCTs assessed had incomplete data and selective report, so these items were appraised as low risk. There is no evidence of other biases, so this item was evaluated as low risk. The detailed quality evaluation of the included studies were shown in **Figures 2** and **3**. Funnel plots were shown in **Figure 4**.

### Efficacy Assessment

#### Clinical Total Effective Rate

**Figure 5:**
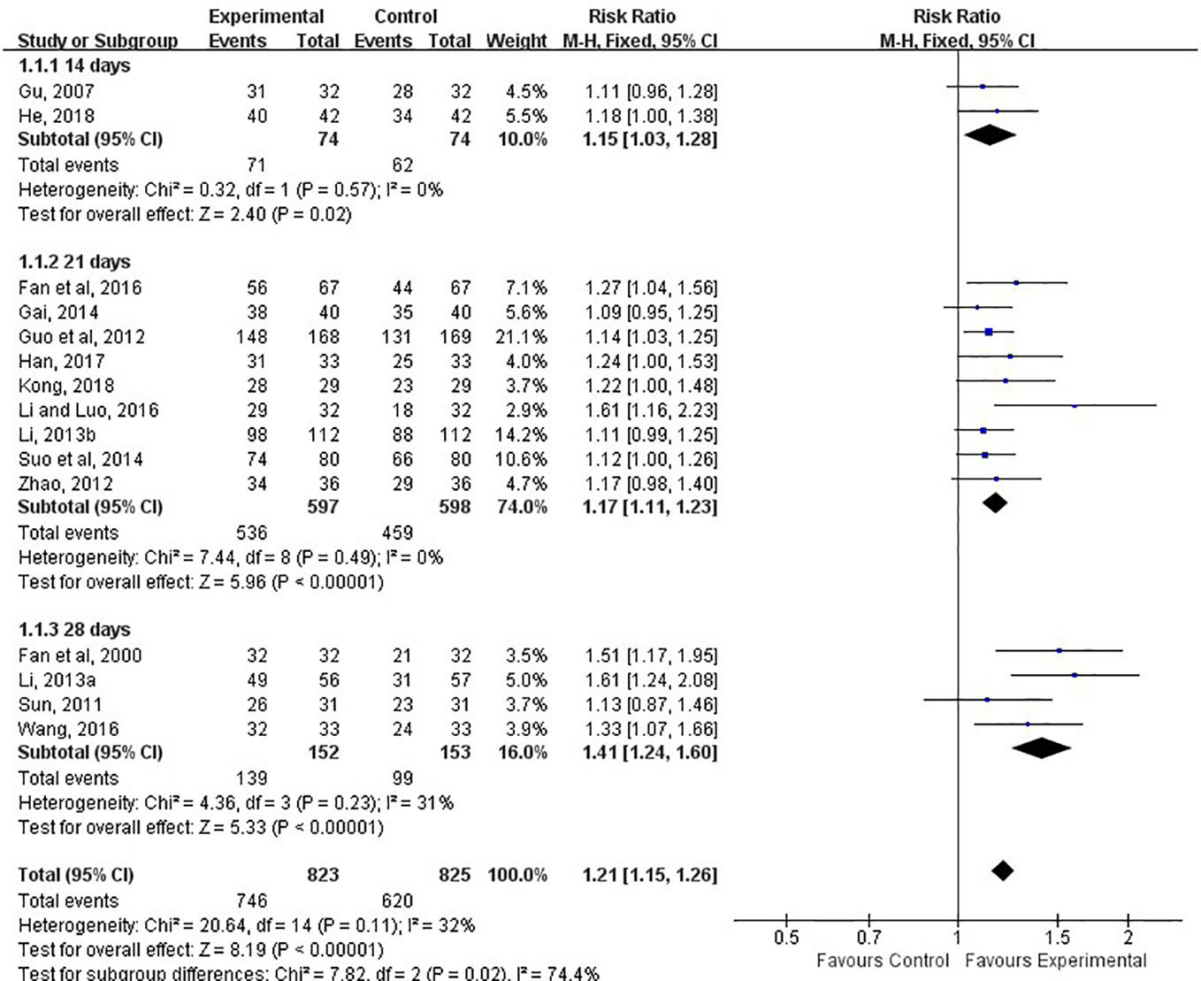
Forest plots showing a improvement of clinical total effective rate in experimental group compared with control group.

The clinical total effective rate was assessed in all included studies. The calculations of all of them was based on the same criteria. It was calculated at 14, 21, and 28 days after treatment. The meta-analysis indicated that the combination of LXTYF and WCM significantly improved the clinical total effective rate compared with WCM alone. The relative ratio (RR) (relative benefit) in 15 studies ranged from 1.09 to 1.61. The overall RR was 1.21 (95% CI, 1.15 to 1.25; *P* < 0.05, *I*^2^ = 32%; **Figure 5**; **Table 3**). In all included studies, Guo et al. had the highest quality and sufficient sample size (Guo et al., 2012). The RR value of their study was 1.14, which was lower than the combined overall RR. Furthermore, the clinical effective rate in experimental group was significantly higher than control group at 14 d (*RR*=1.15, 95%*CI*: 1.03 to 1.28, *P* < 0.05, *I*^2^ = 0%), 21 d (*RR*=1.17, 95%*CI*: 1.11 to 1.23, *P* < 0.05, *I*^2^ = 0%) and 28 d (*RR*=1.41, 95%*CI*: 1.24 to 1.60, *P* < 0.05, *I*^2^ = 31%).

**Table 3:** Summary of Meta-analysis.

Outcome	Studies	Participants	statistical method					
			RR(Fixed), 95%*CI*		OR(Fixed), 95%*CI*		RD(Fixed), 95%*CI*	
			Effect Estimate	*P*	Effect Estimate	*P*	Effect Estimate	*P*
Clinical total effective rate	15	1648	1.21 (1.15,1.26)	<0.00001	3.23 (2.43,4.29)	<0.00001	0.15 (0.12,0.19)	< 0.00001

#### TCM Syndrome Score

**Figure 6:**
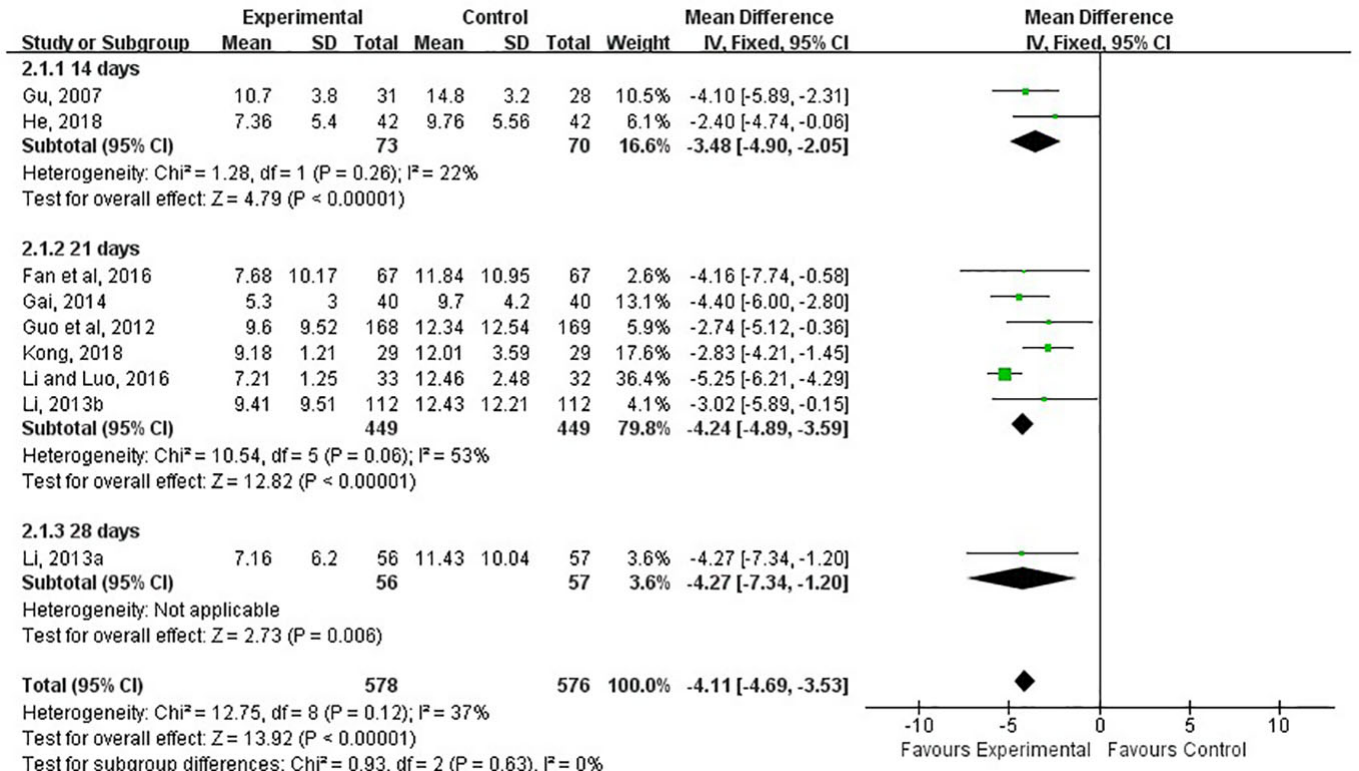
Forest plots showing a reduction of TCM syndrome score in experimental group compared with control group.

The TCM syndrome scoring was reported in 9 of the 15 studies and assessed at 14th, 21st, 28th days after treatment (Gu et al., 2007; Guo et al., 2012; Gai, 2014; Li and Luo, 2016; He, 2018; Kong, 2018; Li L. X. 2013; Li X. 2013; Fan et al., 2016). The meta-analysis showed the combination of LXTYF and WCM prominently decreased the TCM syndrome score compared with the WCM alone (MD, −4.11; 95% CI, −4.69 to −3.53; *P* < 0.05; *I*^2^ = 37%; **Figure 6**). After treatment, remarkable difference between two comparison groups were observed at all evaluation time points (14 d [MD, −3.84; 95% CI, −4.90 to −2.05; *P* < 0.05; *I*^2^ = 22%]; 21 d [MD, −4.24; 95% CI, −4.89 to −3.59; *P* < 0.05; *I*^2^ = 53%]; 28 d [MD, −4.27; 95% CI, −7.34 to −1.20, *P* < 0.05]).

#### Volume of Cerebral Hematoma

**Figure 7:**
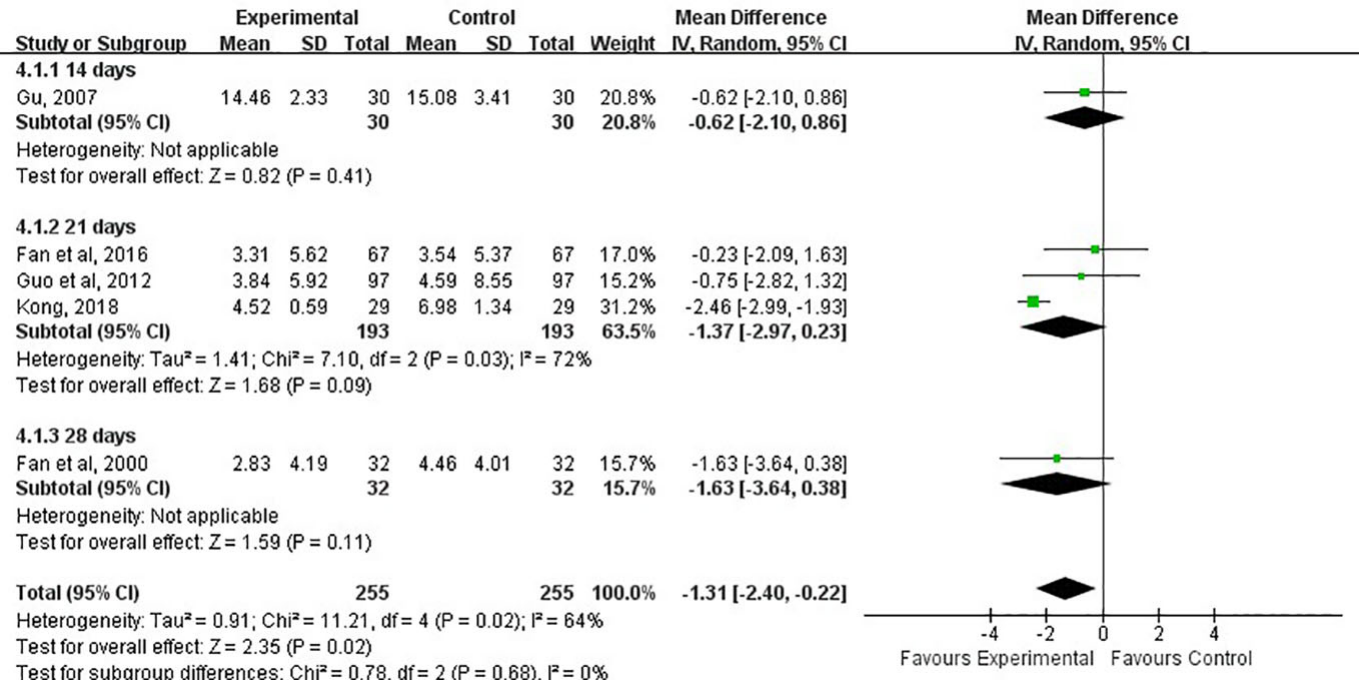
Forest plots showing a reduction of volume of cerebral hematoma in experimental group compared with control group.

The volume of the cerebral hemorrhage after treatment was assessed in five studies (Fan et al., 2000; Gu et al., 2007; Guo et al., 2012; Fan et al., 2016; Kong, 2018). Although the combination of LXTYF and WCM prominently decreased the volume of cerebral hematoma compared with the WCM alone, there were significant differences between two comparison groups (MD, −1.31; 95% CI, −2.40 to −0.22; *P* = 0.02; *I*^2^ = 64%, **Figure 7**).

#### GOS Score Difference

**Figure 8:**
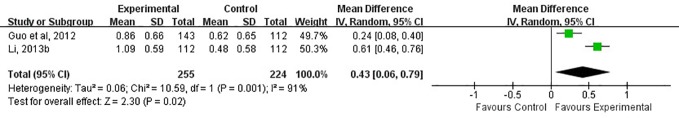
Forest plots showing a reduction of GOS score in experimental group compared with control group.

Glasgow outcome score (GOS) were divided into five levels: good recovery, moderate disability, severe disability, vegetative state, and death. The higher the score, the worse the prognosis. Two of the 13 studies used GOS to reflect the recovery of physical sign and symptoms of neurological deficits in patients with cerebral apoplexy (Guo et al., 2012; Li L. X., 2013). The GOS in two studies were all evaluated at 21st days after treatment. The overall results indicated that the experimental group had a measurably better recovery of neurological functions than the control group (MD, 0.43; 95% CI, 0.06 to 0.79, *P*=0.02, *I*^2^ = 91%, **Figure 8**) with random effect model. Compared with control group, the experimental group showed a greater decrease in GOS score after treatment.

#### ADL Score

**Figure 9:**
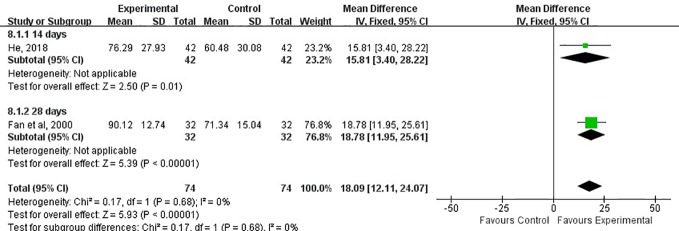
Forest plots showing a improvement of ADL score in experimental group compared with control group.

Activities of daily living (ADL) score refers to daily living ability, which reflects people’s most basic ability in the family(or medical institution) and community. It is the most important index in rehabilitation medicine. The higher the ADL score, the more self-care ability you have. Of 15 RCTs, Only two studies used this index to evaluate the impairment of nerve function. The ADL score in two studies were assessed at 14 and 28 days, respectively (Fan et al., 2000; He, 2018). The results showed that the experiment group had a higher score than the control group (MD, 18.09; 95% CI, 12.11 to 24.07; *P* < 0.05; *I*^2^ = 0%; **Figure 9**).

### Adverse Events

There were three studies (Fan et al., 2000; Gai, 2014; He, 2018) reported adverse events. Fan et al. reported that there were two cases of mild diarrhea and three cases of stomach upset in the experiment group. The symptoms disappeared after taking the LXTYF after the meal. In the He’s report, there were three adverse events in control group and four in experimental group, all of which were mild impairment of liver function and slight elevation of creatine kinase. Under close monitoring, all patients with adverse events did not take targeted drugs and successfully completed the treatment as plan. Gai reported that there was one patient had nausea, and two patients had headache in the control group, while two cases of nausea observed in the experimental group. After stopping the medication, the symptoms relieve spontaneously without no treatment. These reports showed that the addition of LXTYF is safe and reliable, without increasing the burden of liver and kidney functions and binging toxic and side effects.

### Sensitivity Analysis

Through sensitivity analysis, we found that our conclusions were robust regardless of whether one study which treatment was Liangxue Tongyu injection included or not, and our overall estimates of the total effective rate and TCM syndrome score remained unchanged. In addition, we used different analytical models, which also showed that our conclusion was robust. We provided these results of analysis in the appendices (**Appendices 1** and **2**).

## Description of the LXTYF

Nine ingredients were included in the 15 RCTs. The mainly used TCMs were Bubali cornu, Rehmanniae radix, Radix et rhizoma rhei, Acori tatarinowii rhizome, Paeoniae radix rubra, Moutan cortex. Based on these ingredients, Notoginseng radix et rhizome, Persicae semen, Gardeniae fructus were also added in one RCT (Gu et al., 2012).The full and validated botanical names of herbs or constituents were listed in **Table 4**.

**Table 4:** Details of the most commonly used ingredients for LXTYF.

Chinese name	Pharmaceutical name	Species	Family	N/15(%)
Shuiniujiao	*Bubali cornu*	*Bubalus bubalis Linnaeu*	*Bovidae*	15(100%)
Dihuang	*Rehmanniae radix*	*Rehmannia glutinosa Libosch*	*Scrophulariaceae*	15(100%)
Dahuang	*Radix et rhizoma rhei*	*Rheum officinale Bail*	*Polygonaceae*	15(100%)
Shichangpu	*Acori tatarinowii rhizoma*	*Acortw tatarinowii Schott*	*Araceae*	13(87%)
Chishao	*Paeoniae radix rubra*	*Paeonia lactiflora Pall*	*Ranunculaceae*	13(87%)
Mudanpi	*Moutan cortex*	*Paeonia suffruticosa Andr*	*Ranunculaceae*	12(80%)
Sanqi	*Notoginseng radix et rhizoma*	*Parmx notoginseng*	*Araliaceae*	1(7%)
Taoren	*Persicae semen*	*Prunus persica Batsch*	*Rosaceae*	1(7%)
Zhizi	*Gardeniae fructus*	*Gardenia jasminoides Ellis*	*Rubiaceae*	1(7%)

## Discussion

### Summary of Main Results

We included in this systematic review 15 randomized controlled trials of 1648 patients with acute ICH. Except for Gu’s study, which used injection, all other studies used LXTYF orally combined with WCM as the intervention. The meta-analysis indicated that combination of LXTYF and WCM could increase 15% of the clinical total effective rate and reduce 4.11 of TCM syndrome score and 0.42 of GOS score and 1.31 ml of volume of hematoma and 18.09 of ADL score. In terms of safety, adverse events were reported in about 1.3% of participants who received the combination therapy. Unfortunately, reporting of adverse events was incomplete.

### Quality of the Evidence

Overall, of the included 15 RCTs, 8 RCTs reported the method of randomization (Gu et al., 2007; Guo et al., 2012; Gai, 2014; Han, 2017; He, 2018; Li X. 2013; Li L. X. 2013; Fan et al., 2000), 5 reported the generation method of random sequence (Fan et al., 2000; Gu et al., 2007; Guo et al., 2012; Gai, 2014; Han, 2017). Only Guo’s Study reported the allocation concealment. No one studies reported the details of the Blinding to participants or outcome assessors. The largest quantity of study was from Guo’s RCTs, which has the largest sample size and the most standard trials protocol.

### Potential Biases in the Review Process

We prepared a funnel plot for the primary outcome of clinical total effective rate and found asymmetrical funnel distribution, which indicated the possibility of publication bias. In addition, although the main ingredients of LXTYF in included studies were not significantly different, the weight of some ingredients were adjusted according to clinical practice, which would introduce heterogeneity and lead to bias.

### Implications for Practice

The treatment of acute stage of ICH in western medicine is just symptomatic treatment, and there are no specific drugs for absorption of hematoma, not to say it reflects the principle of individualized treatment.

Many studies have showed that TCM can not only improve brain microcirculation, but also promote the absorption of hematoma effectively and improve the prognosis if neurological function. However, due to the lack of multi-centers RCTs, many TCMs have not been widely used. In addition, most of the current clinical guidelines on the treatment of cerebral hemorrhage by TCM are expert consensus and experience, and lack of evidence-based evidence (Gao, 2016).

LXTYF includes eight ingredients and has been used to treat ICH in the clinical. Some pharmacological studies have shown that LXTYF had integrated therapeutic effect on ICH due to activities of anti-inflammatory, anti-coagulation, blood vessel protection, and protection neuron from excitotoxicity. The compound combination in LXYTF, including Taurine, Paeonol, and Ginsenoside Rb1, can offer protection neuron from excitotoxicity at the low concentration by activation of the PI3K/AKT pathway (Li et al., 2018).

In this review, we focused on the clinical efficacy and safety of LXTYF. Through this work, we hope to inherit the experience of famous TCM doctors and to provide evidence-based evidence for traditional Chinses medicine and integrated traditional Chinses and western medicine in treating acute stroke.

### Limitation of the Research

The enrolled patients in RCTs were merely Chinese. Therefore, we could not confirm whether there was a similar effect for non-Chinese. The included studies in this review did not have long-term follow-up data after the end of 14 to 28 days of treatment. Thus, we could not evaluate the long-term effect. In this review, we found that only two RCT reported the GOS and ADL scores, which mainly reflect the neurological impairment. It will not help us to evaluate the real effect of LXTYF in improving prognosis.

## Conclusion

In summary, based on current evidence, we concluded the combined therapy could increase the clinical total effective rate, reduce the degree of neurological deficit, and improve prognosis, and there was no evidence to show that the combination therapy would lead to safety problems. A number of sensitivity analysis have shown that our conclusions are robust. However, considering the potential biases and limitations of our study, additional large, high-quality RCTs are required in the future to confirm or refute the effects of LFTYF combined with WCM in acute stroke.

## Author Contributions

GL and JD designed the study. CJ and XY contributed to data collection and statistical analysis. Results were interpreted by GL and CJ. The report was drafted by CJ. Critical revision of the report for important intellectual content was done by all the investigators.

## Funding

This study was funded by the Jiangsu Natural Science Foundation (No. SBK2016022369) and the Priority Academic Program Development of Jiangsu Higher Education Institutions (integration of Chinese and Western medicine).

## Conflict of Interest

The authors declare that the research was conducted in the absence of any commercial or financial relationships that could be construed as a potential conflict of interest.

## Appendix 1 Summary of sensitivity analysis (continuous outcomes).

**Table:**

Outcome	time	studies	Participants	statistical method					
				MD, 95%CI			SMD, 95%CI		
				Effect Estimate	*P*	Model	Effect Estimate	*P*	Model
TCM syndrome score	Baseline	9	1159	−0.18 (−1.11 to 0.75)	0.71	Fixed	−0.02 (−0.13 to 0.10)	0.77	Fixed
	Visit	9	1154	−4.11 (−4.69 to −3.53)	< 0.00001	Fixed	−0.81 (−1.17 to −0.45)	< 0.0001	Random
Volume of hematoma	Baseline	5	645	0.33 (−1.03 to 1.70)	0.63	Fixed	−0.02 (−0.17,0.14)	0.84	Fixed
	Visit	5	510	−1.31 (−2.40 to −0.22)	0.02	Random	−0.56 (−1.16 to 0.03)	0.06	Random
GOS	Diff	2	511	0.42 (0.06–0.79)	0.02	Random	0.70 (0.04–1.36)	0.04	Random
ADL	Baseline	2	148	0.50 (−4.09 to 5.09)	0.83	Fixed	0.04 (−0.29 to 0.36)	0.83	Fixed
	Visit	2	148	18.09 (12.11–24.07)	< 0.00001	Fixed	0.92 (0.14–1.69)	0.02	Random

## Appendix 2 Summary of sensitivity analysis (categories outcome).

**Table:**

Outcome	Studies	Participants	statistical method					
			RR(Fixed), 95%CI		OR(Fixed), 95%CI		RD(Fixed), 95%CI	
			Effect Estimate	*P*	Effect Estimate	*P*	Effect Estimate	*P*
Clinical total effective rate	15	1648	1.21 (1.15,1.26)	< 0.00001	3.23 (2.43,4.29)	< 0.00001	0.15 (0.12,0.19)	< 0.00001
