# Supplementary material for: Systematic Review and Meta-Analysis of Randomized Controlled Trials of Liangxue Tongyu Formula on Patients With Acute Intracerebral Hemorrhage
Source: Front Pharmacol. 2020 Apr 15;11:437. doi: 10.3389/fphar.2020.00437 (PMC7174629; doi:10.3389/fphar.2020.00437)
Supplement: Supplementary file 1 [file DataSheet_1.pdf]

GC-MS experiment

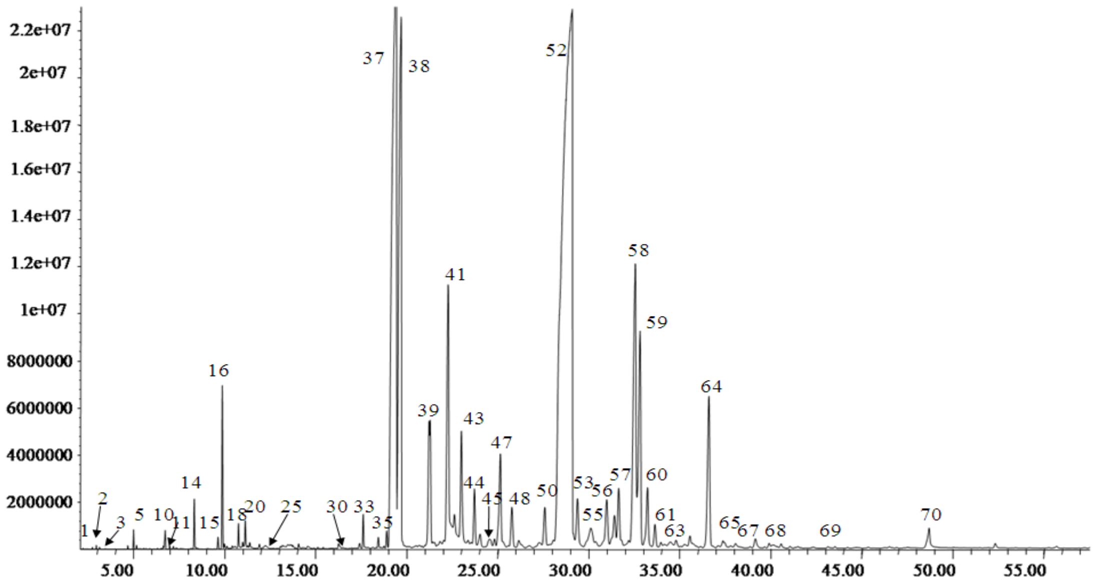

Figure S1 Total ion current map of volatile oil part of LTP

Table S1 Chemical components of volatile oil from LTP

No.	*t*/min	Compound	*Content* (%)
1	2.76	Hexanal	0.01
2	3.15	Furfural	0.01
3	4.51	1,7,7-Trimethyl-tricyclo[2.2.1.0(2,6)]heptane	0.01
4	4.70	α-Pinene	0.01
5	5.01	Camphene	0.08
6	5.18	Benzaldehyde	0.02
7	5.42	4-Methylene-1-(1-methylethyl)-bicyclo[3.1.0]hexane	0.01
8	5.56	β-Pinene	0.01
9	6.55	1-Methyl-3-(1-methylethyl)-benzene	0.01
10	6.75	Eucalyptol	0.13
11	6.99	Benzeneacetaldehyde	0.02
12	7.20	Dodecane	0.04
13	7.36	1-Methyl-4-(1-methylethyl)-1,4-cyclohexadiene	0.02
14	8.35	3,7-Dimethyl-1,6-octadien-3-ol	0.28
15	9.65	6,6-Dimethyl-bicyclo[3.1.1]heptan-2-one	0.07
16	9.88	(1*R*)-1,7,7-Trimethyl-bicyclo[2.2.1]heptan-2-one	0.93
17	10.00	2,3,3-Trimethyl-bicyclo[2.2.1]heptan-2-ol	0.03
18	10.77	Terpinen-4-ol	0.16
19	11.01	Myrtanal	0.07
20	11.14	α-4-Trimethyl-3-cyclohexene-1-methanol	0.18
21	11.29	2-Hydroxy-benzoic acid methyl ester	0.03

22	11.39	(1*R*)-(-)-Myrtenal	0.09
23	11.92	β-Methyl-cinnamaldehyde	0.05
24	12.24	(*R*)- 3,7-Dimethyl-6-octen-1-ol	0.10
25	12.68	2-Methyl-3-phenyl-propanal	0.01
26	13.49	6,6-Dimethyl-bicyclo[3.1.1]heptane-2-methanol	0.08
27	14.08	Acetic acid,1,7,7-trimethyl-bicyclo[2.2.1]hept-2-ylester	0.05
28	14.30	Tridecane	0.03
29	14.59	4-(1-Methylethenyl)-1-cyclohexene-1-methanol	0.06
30	16.27	2-Methoxy-3-(2-propenyl)-phenol	0.07
31	16.99	α-Cubebene	0.03
32	17.43	[1*S*-(1α,2β,4β)]-1-Ethenyl-1-methyl-2,4-*bis*(1-methylethenyl)-cyclohexane	0.06
33	17.62	1,2-Dimethoxy-4-(2-propenyl)-benzene	0.24
34	18.15	β-Selinene	0.03
35	18.45	Aristolene	0.12
36	18.91	[1a*R*-(1aα,7α,7aα,7bα)]-1a,2,3,5,6,7,7a,7b-Octahydro -1,1,7,7a-tetramethyl-1*H*-cyclopropa[a]naphthalene	0.13
37	19.41	Paeonol	15.76
38	19.68	1,2-Dimethoxy-4-(1-propenyl)-benzene	9.47
39	21.80	1-(2,6,6-Trimethyl-1-cyclohexen-1-yl)-1-penten-3-one	0.08
40	22.01	Butylated hydroxytoluene	0.06
41	22.26	Shyobunone	4.04
42	22.60	(1*S*-*cis*)-1,2,3,5,6,8a-Hexahydro-4,7-dimethyl-1-(1-methylethyl)-naphthalene	0.75
43	22.98	Octahydro-4,4,8,8-tetramethyl-4a,7-methano-4a*H*-naphth[1,8a-b]oxirene	1.62
44	24.00	1,2,3-Trimethoxy-5-(2-propenyl)-benzene	0.28
45	24.52	7-(1-Methylethylidene)-bicyclo[4.1.0]heptane	0.29
46	24.80	1,2-Dihydro-1,1,6-trimethyl-naphthalene	0.14
47	25.11	1,2-Dimethoxy-4-(2-methoxy-1-propenyl)benzene	1.49
48	25.75	[1aγ-(1aα,4aα,7β,7aβ,7bα)]-Decahydro-1,1,7-trimethyl-4-methylene-, 1*H*-cycloprop[e]azulen-7-ol	0.60
49	26.13	Isoaromadendrene epoxide	0.23
50	27.56	Dehydroxy-isocalamendiol	0.63
51	28.04	[1a*R*-(1aα,4α,4aβ,7bα)]-1a,2,3,4,4a,5,6,7b-Octahydro -1,1,4,7-tetramethyl-1*H*-cycloprop[e]azulene	0.22
52	29.06	β-Asarone	36.88
53	29.36	Dehydroxy-isocalamendiol	0.84

54	30.09	2-Isopropyl-5-methyl-9-methylene-bicyclo[4.4.0]dec -1-ene	0.73
55	30.35	Copaene	0.15
56	30.96	α-Cadinol	0.96
57	32.17	1,6-Dimethyl-4-(1-methylethyl)-naphthalene	0.19
58	32.54	α-Asarone	5.41
59	32.79	Zierone	3.37
60	33.20	1,7-Dimethyl-4-(1-methylethyl)-spiro[4.5]dec-6-en-8-one	0.99
61	33.61	3,5,6,7,8,8a-Hexahydro-4,8a-dimethyl-6-(1-methylethenyl)-2(1*H*)naphthalenone	0.36
62	33.94	1,2,3,4-Tetrahydro-3-isopropyl-5-methyl-1-oxonaphthalene	0.06
63	34.77	2,4,5-Trimethoxy-benzaldehyde	0.17
64	36.57	4-Acetyl-7,7-dimethyl-2-(2-oxopropyl)-cycloheptanone	2.46
65	37.35	Tetradecanoic acid	0.21
66	38.04	6-Isopropenyl-4,8a-dimethyl-1,2,3,5,6,7,8,8a-octahydronaphthalene-2,3-diol	0.16
67	39.13	6-(1-Hydroxymethylvinyl)-4,8a-dimethyl-3,5,6,7,8,8a-hexahydro-1*H*-naphthalen-2-one	0.21
68	39.89	α-Isobutyl-2,4,5-trimethyl-benzyl alcohol	0.08
69	43.12	Pentadecanoic acid	0.04
70	48.67	*n*-Hexadecanoic acid	0.39

HPLC-MS experiment

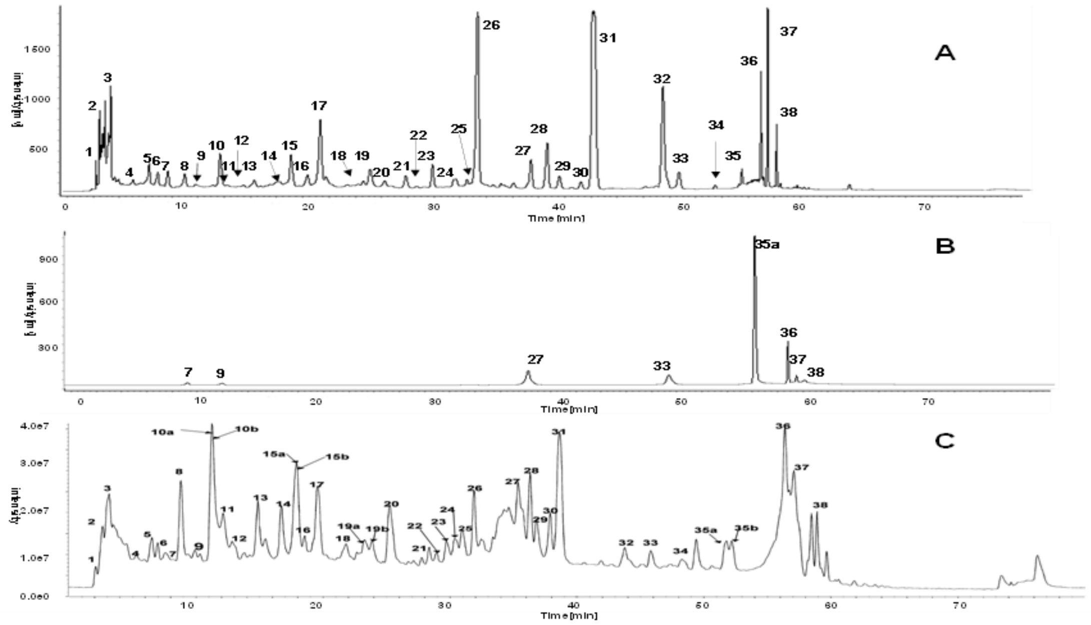

Figure S2 HPLC chromatogram of *n*-butanol extract from LTP (A) and standards

solution (B) and TIC of *n*-butanol extract from LTP (C)

Table S2 Results of the constituents of MS-ESI

No.	*t*/min	MS	MS/MS	MW	Compound
1	2.69	169	125,97,81	170	Gallic acid
2	3.18	463	433,403,343,301,241,169,125	464	Mudanoside B
3	4.07	361	361,343,199	362	Catalpol
4	4.49	407	347,329,185,167,147,127,59	348	Leonuride
5	6.53	495	465,281,165,137	496	Oxypaeoniflorin
6	6.86	183	124,95,78	184	Methyl gallate
7	7.98	539	449,435,357,283,195,121	480	Albiflorin
8	9.12	525	495,345,329,311,177,165,123	526	Mudanpioside E
9	10.53	539	449,327,165,121	480	Paeoniflorin
10a	11.29	647	509,399,313,271,211	648	Galloyl-oxypaeoniflorin
10b	11.53	635	483,465,313,295,271,169,125	636	Trigalloyl glucose
11	12.16	787	635,617,483,465,277,169,125	788	Tetragalloyl glucose
12	12.81	611	445,343,301,283,169,151,125	612	Suffruiticoside AorC
13	15.54	611	445,343,313,283,169,151,125	612	Suffruiticoside BorD
14	16.77	631	613,491,399,313,271,169,125	632	Galloyl-paeoniflorin
15a	17.01	335	183,169,124,95	336	Galloyl methyl gallate
15b	17.87	939	769,393,169,317,617	940	Pentagalloyl glucose
16	18.56	623	461,315,179,161,133	624	Resveratrol galloyl glucoside
17	19.44	479	433,357,327,283,195,121	480	Mudanpioside I
18	22.62	615	615,585,477,281,239	616	iso-Mudanpioside H
19a	23.22	541	313,227,169,125	542	Resveratrol galloyl glucoside
19b	23.95	1091	939,769,617,447,169	1092	Hexagalloyl glucose
20	25.09	571	571,313,257,241,169	572	Unidentied
21	29.61	599	551,447,477	600	Benzoyloxypaeoniflorin
22	29.82	629	599,507,167,121	630	Mudanpioside J
23	30.16	461	461,313,169,125	462	Cinnyl galloyl glucoside
24	30.28	165	150,135,122,91,65	166	Paeonol
25	30.97	599	569,477,403,447,333	600	Mudanpioside C
26	31.92	431	269,241,240,225	432	Aloeemodin-glucoside
27	35.31	643	553,535,431,265,165,121	584	Benzoylpaeoniflorin
28	35.96	415	277,253,225	416	Chrysophanol-glucoside
29	36.88	991	931,799,637,475	932	Notoginsenoside- R_1
30	37.91	431	269,241,225	432	Emodin glucoside
31	38.65	859	799,637,475	800	Ginsenoside- Rg_1
32	43.49	607	443,295,169,125	608	4-Hydroxyphenyl-2-butanone cinnamic acid, galloyl glucoside
33	45.81	269	240,223	270	Aloeemodin

34	48.41	859	859,619	800	Ginsenoside-Rf
35a	51.58	283	257,239,211,183	284	Rhein
35b	52.19	697	637,475	638	Ginsenoside- Rh_1
36	56.78	269	241,225	270	Emodin
37	57.46	253	239,225	254	Chrysophanol
38	59.64	283	225,183	284	Physcion
